# Heat shock protein 70-mediated sensitization of cells to apoptosis by Carboxyl-Terminal Modulator Protein

**DOI:** 10.1186/1471-2121-10-53

**Published:** 2009-07-15

**Authors:** Longzhen Piao, Yuwen Li, Keum-Jin Yang, Kyeong Ah Park, Hee Sun Byun, Minho Won, Janghee Hong, Jeong-Lan Kim, Gi Ryang Kweon, Gang Min Hur, Jeong Ho Seok, Jae Youl Cho, Taehoon Chun, Daniel Hess, Ragna Sack, Sauveur-Michel Maira, Derek P Brazil, Brian A Hemmings, Jongsun Park

**Affiliations:** 1 Department of Pharmacology, Daejeon Regional Cancer Center, Cancer Research Institute, Research Institute for Medical Sciences, College of Medicine, Chungnam National University, Taejeon, 301-131, Korea; 2 Department of Psychiatry, College of Medicine, Chungnam National University, Taejeon, 301-131, Korea; 3 Department of Biochemistry, College of Medicine, Chungnam National University, Taejeon, 301-131, Korea; 4 School of Bioscience and Biotechnology, and Institute of Bioscience and Biotechnology, Kangwon National University, Chuncheon, 200-701, Korea; 5 Division of Biotechnology, School of Life Sciences and Biotechnology, Korea University, Seoul, 136-701, Korea; 6 Friedrich Miescher Institute, Maulbeerstrasse 66, CH-4058 Basel, Switzerland; 7 Novartis Institutes for Biomedical Research, Oncology Disease Area, Novartis Pharma AG, CH4002 Basel, Switzerland; 8 UCD Diabetes Research Centre, UCD School of Biomolecular and Biomedical Science, UCD Conway Institute, University College Dublin, Belfield, Dublin 4, Ireland; 9 Department of Internal Oncology, Affiliated Hospital of Yanbian University College of Medicine, Yanji 133000, Jilin Province, PR China

## Abstract

**Background:**

The serine/threonine protein kinase B (PKB/Akt) is involved in insulin signaling, cellular survival, and transformation. Carboxyl-terminal modulator protein (CTMP) has been identified as a novel PKB binding partner in a yeast two-hybrid screen, and appears to be a negative PKB regulator with tumor suppressor-like properties. In the present study we investigate novel mechanisms by which CTMP plays a role in apoptosis process.

**Results:**

CTMP is localized to mitochondria. Furthermore, CTMP becomes phosphorylated following the treatment of cells with pervanadate, an insulin-mimetic. Two serine residues (Ser37 and Ser38) were identified as novel *in vivo *phosphorylation sites of CTMP. Association of CTMP and heat shock protein 70 (Hsp70) inhibits the formation of complexes containing apoptotic protease activating factor 1 and Hsp70. Overexpression of CTMP increased the sensitivity of cells to apoptosis, most likely due to the inhibition of Hsp70 function.

**Conclusion:**

Our data suggest that phosphorylation on Ser37/Ser38 of CTMP is important for the prevention of mitochondrial localization of CTMP, eventually leading to cell death by binding to Hsp70. In addition to its role in PKB inhibition, CTMP may therefore play a key role in mitochondria-mediated apoptosis by localizing to mitochondria.

## Background

Protein kinase B (PKB/Akt) is activated by receptor tyrosine kinases and regulates cell proliferation, survival, and motility [1,2]. PKB activation occurs when PtdIns[3-5]P_3 _(a product of PI3K) binds to the pleckstrin homology domain of PKB. Phosphorylation of two amino acids (Thr308 and Ser473) is then required for full PKB activation [3,4]. Unphosphorylated PKB is inactive, but PKB phosphorylation on Thr308 (by PDK1) stimulates PKB activity by approximately 100-fold [2]. Phosphorylation on a second regulatory site at the carboxyl terminus (Ser473; termed the hydrophobic motif) by rictor-mTOR and DNA-PK can further activate PKB seven- to ten-fold ([5-7]. The crystal structure of PKB suggests that Ser473 phosphorylation is important for kinase activation and stabilization [8,9].

Recently, C-terminal modulator protein (CTMP) was identified as a PKB binding partner [10]. CTMP overexpression inactivates PKB in v-Akt-transformed cells transplanted into mice [10], in cultured cells [11], and in a K-ras-induced lung cancer model [12]. The tumor suppressor-like properties of CTMP are supported by a report demonstrating inhibition of CTMP expression by hypermethylation of its promoter in malignant glioblastomas, where PKB activity is frequently altered [13].

Mitochondria regulate cellular energy supplies, apoptosis, and signaling pathways. Alterations in mitochondrial function are responsible for a range of inherited and acquired human diseases and are implicated in the aging process [14]. Cytosolic mitochondrial protein precursors contain information that is necessary to direct them to the mitochondria. Mitochondrial precursor proteins in the cytosol are present as complexes with factors that stabilize them, as they are prone to degradation and aggregation. Several such factors are implicated as cytosolic chaperones; however, convincing data exist only for heat shock protein 70 (Hsp70) and Hsp90 [15,16].

Here, we describe CTMP phosphorylation on two sites following treatment with pervanadate, an insulin-mimetic. Surprisingly, CTMP C-terminally tagged with GFP was localized to mitochondria, whereas CTMP N-terminally tagged with GFP was mainly found in the cytoplasm. Consistent with this observation, mitochondrial localization of endogenous and exogenous CTMP has recently been reported [17] while this study was in review. Mitochondrial localization of CTMP was dependent on an N-terminal mitochondrial targeting sequence (MTS) and was inhibited by phosphorylation on Ser37/Ser38. Finally, CTMP overexpression sensitizes the cell to apoptosis by sequestering Hsp70 away from apoptotic protease activating factor 1 (Apaf-1), suggesting that CTMP is involved in apoptotic processes through its mitochondrial localization and binding to Hsp70.

## Results

### Identification of phosphorylated residues of CTMP in pervanadate-stimulated CCL64 cells

**Figure 1:**
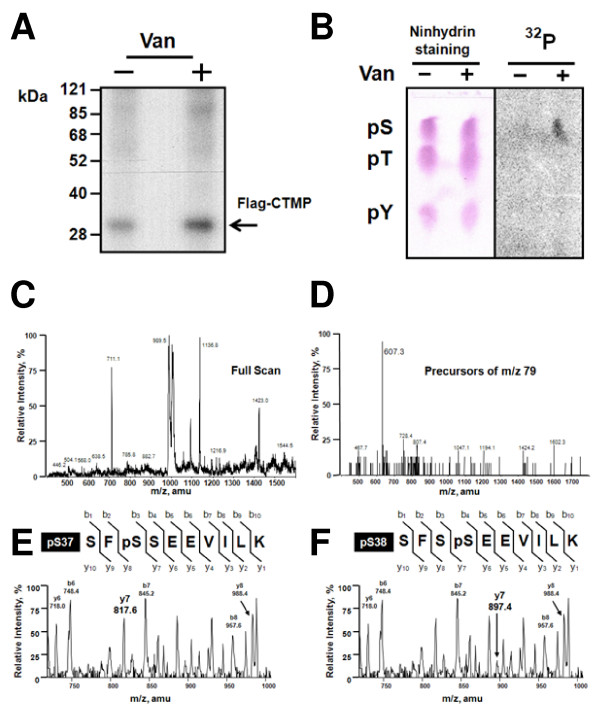
**Serine 37 or Serine 38 of CTMP is phosphorylated *in vivo *following stimulation of CCL64 cells with pervanadate**. CCL64 cells expressing Flag-CTMP were metabolically labeled with ^32^P_i _prior to pervanadate treatment. (A) Immunoprecipitated ^32^P-labeled CTMP and (B) Extracted protein from both conditions was hydrolyzed and analyzed as described in Experimental Procedures. (Phosphoserine: pS, phosphothreonine: pT, and phosphotyrosine: pY) (C) Full scan mass spectrum of fraction 16 in the positive mode was acquired. (D) *m/z* values of the phosphopeptides were identified by *m/z* 79 precursor-ion scanning in the negative ion mode. (E and F). Tandem mass spectra derived by CID of the (M + 2H) 2+ precursor, *m/z* 609.3. Fragment ions in the spectra represent many single-event preferential cleavages of peptide bonds, resulting in the sequence information recorded from both the N- and C-termini of the peptide simultaneously. The single-letter code for the amino acids is shown.

We previously observed CTMP phosphorylation upon pervanadate treatment in cells stably expressing Flag-CTMP [10], prompting us to map the phosphorylation sites in CTMP. Flag-CTMP was isolated from ^32^P-metabolically labeled cells prior to pervanadate treatment. Autoradiography after SDS-PAGE showed that CTMP phosphorylation was higher in pervanadate-treated cells versus untreated (Figure 1A). Phosphoamino acid analysis of total protein revealed this 3-fold increase of ^32^P-incorporation was predominantly due to serine phosphorylation (Figure 1B). Bands corresponding to Flag-CTMP were excised and digested with trypsin. The resultant mixture of peptides was separated on a C_18 _column by HPLC. The UV-trace, as well as the peptide masses detected was very similar between two samples whereas the radiolabeled phosphopeptide abundance derived from pervanadate treated cells was markedly increased compared to control cells (data not shown). Fractions with higher radioactivity were further analyzed by NanoESI-MS/MS [18]. The NanoESI-MS/MS measures the mass to charge ratio (*m/z*) of each peptide in the mixture that, upon fragmentation, liberates a species with a *m/z* of 79 Da (corresponding to a single phosphate group) allowing detection of phosphopeptides among other peptides in each fraction. An example of the specificity is shown for fraction 16 from pervanadate-treated cells (Figure 1D). Approximately 20 peptides were detected by NanoESI-MS in the positive full scan mode (Figure 1C); however, only one phosphopeptide was detected in the *m/z* 79 precursor scan mode (Figure 1D). The observed *m/z* value of 607 accounted for the CTMP-derived peptide SF SSEEVILK (35–44 a.a.), which was 80 Da heavier than expected for the non-phosphorylated form based on phosphorylation at one residue. To precisely locate the phosphorylation site of this phosphopeptide, CID tandem MS was performed [19]. The CID tandem MS clearly showed the phosphate was located on either Ser37 or Ser38, but not at both residues, based on the observed mass (1218 Da). Since we detected the ions y_6 _(m/z 730), y_7 _(m/z 817), (indicating Ser37 was phosphorylated (Figure 1E)), as well as the ions y_6 _(*m/z* 730), y_7 _(*m/z* 897), and y_7_-H_3_PO_4_ (*m/z*799), (suggesting Ser38 was phosphorylated (Figure 1F)), we concluded this fraction probably contained a mixture of the peptide phosphorylated at either Ser37 or Ser38.

### CTMP is localized to the mitochondrial intermembrane space and/or matrix

**Figure 2:**
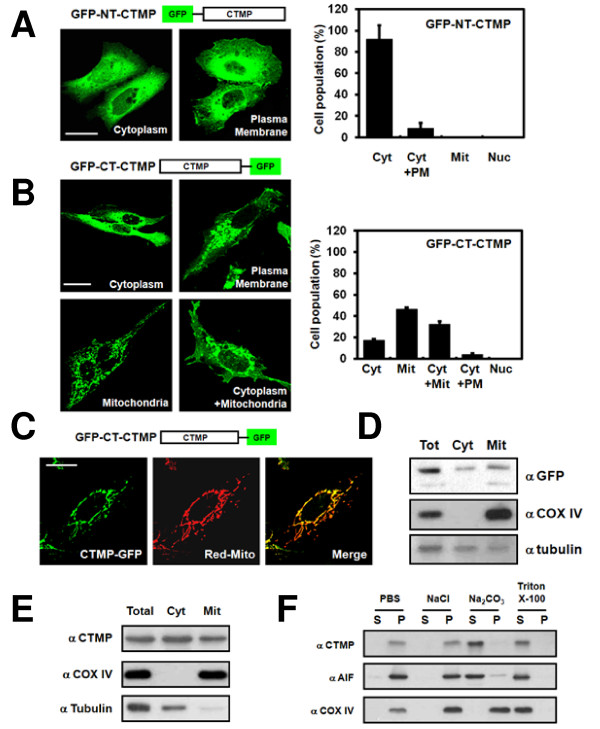
**Functional mitochondrial localization of CTMP**. U2OS cells were transfected with (A) CTMP GFP-tagged at the N-terminus (GFP-NT-CTMP) or (B) CTMP GFP-tagged at the C-terminus (GFP-CT-CTMP) for 24 h. Differential localization of CTMP (Cyt: cytoplasm, PM: plasma membrane, Mit: mitochondria, Nuc: Nucleus) was examined using confocal microscopy. At least 200 cells were counted from three distinct fields for each transfected group. U2OS cells were co-transfected with (C) pEGFP-N3-CTMP and pDsRed-mito for 24 h and analyzed by confocal microscopy. (D) U2OS cells were transfected with pEGFP-N3-CTMP. Total lysates (Tot), cytosolic fractions (Cyt), and mitochondrial fractions (Mit) were analyzed by immunoblot analysis. (E) The subcellular fractions of HEK 293 cells were analyzed with the indicated antibodies. (COX IV, Mit marker; α-tubulin, Cyt marker). (F) Mitochondrial fractions of HEK 293 cells were isolated and treated under the indicated conditions. Samples were separated into the supernatant (S) and precipitate (P) fractions and then analyzed. (AIF, Mit intermembrane space marker).

We previously reported that CTMP localized to the plasma membrane, leading to PKB inhibition [10]. Analysis of the CTMP sequence using the PSORTII prediction algorithm [20] indicated CTMP had a 69.6% probability for mitochondrial localization with a 21.7% probability for cytoplasm localization. These findings were also supported by TargetP V1.0 [21] and MitoProt II 1.0a4 [22]. Therefore, CTMP subcellular localization in U2OS cells was examined using GFP-NT-CTMP [10], since U2OS cells exhibit epithelial adherent morphology and are convenient for localization studies. Confocal imaging analysis revealed that 92% of cells expressed GFP-NT-CTMP in the cytoplasm, with a smaller amount of cells (8%) expressing GFP-NT-CTMP at the plasma membrane (Figure 2A). GFP C-terminal tagged CTMP were also prepared to explore the possibility that the GFP tag at the N-terminus affected mitochondrial localization of CTMP [23]. Strikingly, about 46% of cells expressed GFP-CT-CTMP in the mitochondria, with 32% of cells expressing GFP-CT-CTMP at the mitochondria and cytoplasm (Figure 2B), indicating that CTMP may localize to the mitochondria. We confirmed these findings using DsRed-mito as a mitochondrial marker, which co-localized with GFT-CT-CTMP (Figure 2C). Additional biochemical analysis, using cell fractionation, indicated CTMP was present in both the mitochondria and cytoplasm (Figure 2D). Since all experiments to date were performed using an overexpression system, we examined the subcellular localization of endogenous CTMP in HEK293 cells. Immunoblot analysis confirmed endogenous CTMP was localized at the mitochondria as well as in the cytoplasm (Figure 2E). To determine the precise localization of CTMP in mitochondria, we first isolated mitochondria fractions which were isolated under the following conditions: i) 2 M NaCl for mitochondria outer membrane, ii) 100 mM Na_2_CO_3_for intermembrane space and/or mitochondrial matrix and iii) 1% (v/v) Triton X-100 for mitochondria inner or outer membrane protein. CTMP was solubilized in Na_2_CO_3 _(Figure 2F), indicating that CTMP is a soluble protein in either the inter-membrane space and/or the mitochondrial matrix.

### Mitochondrial targeting sequence-mediated mitochondrial localization of CTMP is inhibited by phosphorylation event

**Figure 3:**
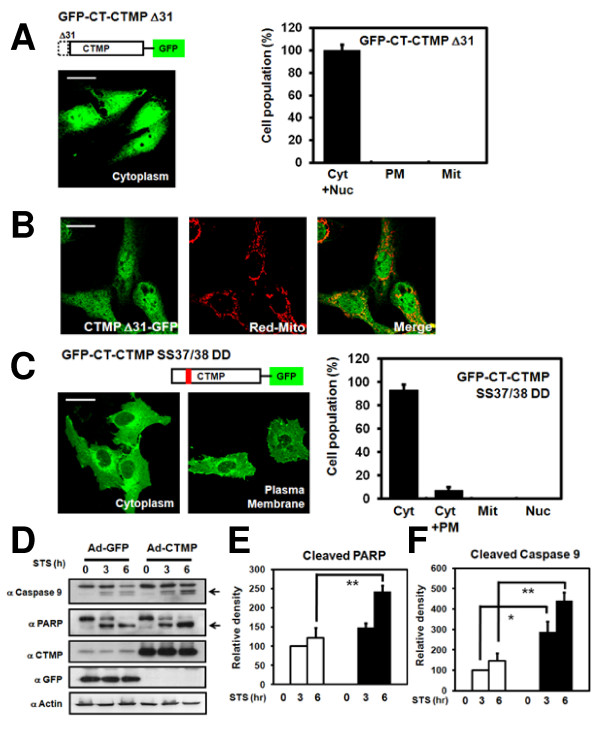
**MTS- and phosphorylation-dependent mitochondria localization of CTMP in U2OS cells**. U2OS cells were transfected with (A) An N-terminal deletion mutant of CTMP (GFP-CT-CTMP ΔN31) or (C) The S37D/S38D negatively charged side group-mimic mutant form of CTMP (GFP-CT-CTMP-S37D/S38D) for 24 h. Differential localization of CTMP (Cyt: cytoplasm, PM: plasma membrane, Mit: mitochondria, Nuc: Nucleus) was examined using confocal microscopy. At least 200 cells were counted from three distinct fields for each transfected group. U2OS cells were co-transfected with (B) pEGFP-N3-Δ31-CTMP and pDsRed-mito for 24 h and analyzed. (D) HeLa cells were transduced with the indicated virus for 24 h, treated with 1 uM staurosporine for 3 and 6 h, harvested, and analyzed. Results are representative of three independent experiments. (E, F) Bands of cleaved PARP and caspase 9 were further analyzed by densitometry. Statistical differences of cleaved protein were determined by comparing values for actin at each lane. The results are mean ± SD of three independent experiments. Asterisk, p < 0.05; double asterisk, p < 0.01.

Bioinformatics analysis of CTMP sequence using MitoProt II 1.0a4 [22] predicted the mitochondrial signal peptide could be cleaved at amino acid position 32, closed to the identified phosphorylation site. In order to investigate the potential mitochondrial targeting sequence (MTS) of CTMP, an N-terminal 31-amino acid deletion-mutant of CTMP (GFP-CT-CTMP ΔN31) was constructed based on the prediction of MitoProt II 1.0a4 [22]. As predicted, this mutant form of CTMP did not localize to the mitochondria (Figure 3A and 3B). Since Ser37 and Ser38 of CTMP were identified as *in vivo *phosphorylation sites (Figure 1E and 1F), a negatively charged side group-mimic CTMP mutant (GFP-CT-CTMP S37D/S38D) was generated. Confocal analysis of cells expressing this negatively charged side group-mimic CTMP mutant showed that the majority of cells expressed CTMP in the cytoplasm (Figure 3C; 93% cytoplasm and 7% cytoplasm and plasma membrane), suggesting phosphorylation on Ser37/Ser38 is an important regulatory mechanism for CTMP shuttling to the mitochondria.

### CTMP overexpression sensitizes the cell to apoptosis induced by staurosporine

To evaluate the apoptotic role of CTMP, we overexpressed CTMP in HeLa cells for 24 h and subsequently treated the cells with 1 μM staurosporine for the indicated times. Stauroporine-mediated apoptosis (assessed by PARP cleavage and Caspase 3/9 activation) was detected at 3 h of treatment (Figure 3D–3F, data not shown). Apoptosis was more pronounced in CTMP-transduced compared to controls, suggesting that CTMP overexpression increases the sensitivity of cells to programmed cell death.

### CTMP-mediated Hsp70 sequestration leads to the dissociation of Hsp70 and Apaf-1

**Figure 4:**
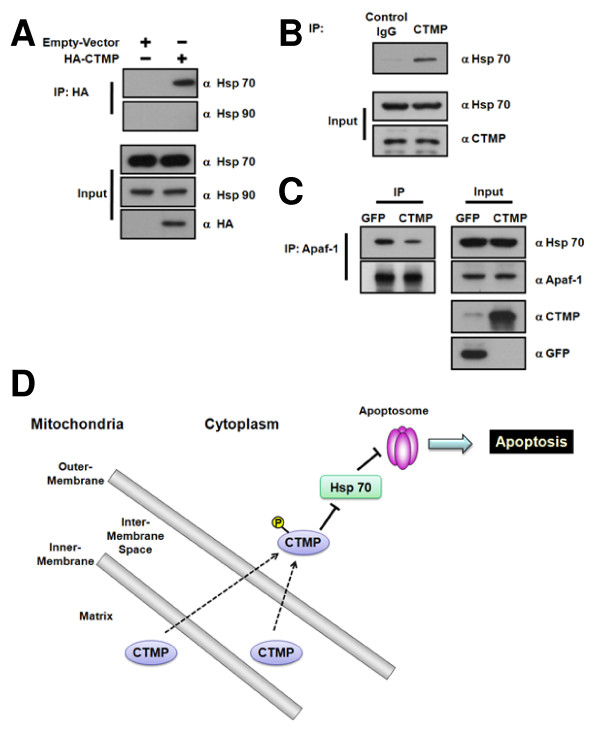
**Binding of CTMP to Hsp70 inhibits the formation of a complex containing Hsp70 and Apaf-1 in HeLa cells**. (A) HeLa cells were transiently transfected with HA-CTMP and cultured for 24 h. Bound proteins in HA-CTMP immunoprecipitates were detected using anti-Hsp70 and anti-Hsp90 antibodies (top two panels). (B) HeLa cell extracts were incubated with anti-CTMP antibody and bound Hsp70 was assessed by immunoblotting (top panel). (C) HeLa cells were infected with GFP- and CTMP-adenovirus for 24 h. After immunoprecipitation of Apaf-1, bound Hsp70 was detected (top panel). Input of each protein was also detected (bottom). Similar results were obtained in three separate experiments. (D) In addition to CTMP's role in PKB inhibition, CTMP has a key role in mitochondria-mediated apoptosis by localizing to mitochondria. CTMP appeared to be phosphorylated on Ser37/Ser38 in response to pervanadate stimulation, resulting in the cytosolic localization of CTMP. In this context, CTMP sensitizes the cell to apoptosis by binding to Hsp70, thus preventing its binding to Apaf-1. Therefore, CTMP may have distinct roles in various subcellular compartments.

Recent studies show that heat-shock proteins (Hsps) family, including Hsp90, Hsp70 and Hsp27, can influence apoptosis through direct physical interaction with key components of the apoptotic machinery [24]. Since CTMP overexpression appears to enhance the staurosporine-induced apoptosis (Figure 3D–3F), the possible interaction of CTMP with these Hsp proteins was monitored in HeLa cells expressing HA-CTMP. Immunoprecipitation of HA-CTMP revealed that Hsp70 interacts with CTMP while Hsp90 displayed negligible binding with CTMP (Figure 4A), suggesting that this binding of CTMP and Hsp70 is specific. Furthermore, endogenous association of CTMP and Hsp70 was also observed (Figure 4B). Since Hsp70 is able to directly inhibit caspase processing by interacting with Apaf-1 to prevent the recruitment of procaspase-9 to the apoptosome [25,26], the interaction between Hsp70 and Apaf-1 was monitored in HeLa cells. The association of Hsp70 to Apaf-1 is significantly reduced in CTMP-infected cells compared to control (Figure 4C). Taken together, CTMP is able to enhance apoptosis process by sequestering Hsp70 thus preventing its binding to Apaf-1.

## Discussion

Mitochondria are essential organelles in most eukaryotic cells and function in the maintenance of cellular energy supplies. Mitochondria are responsible for thermoregulation and synthesis of essential molecules [14]. In addition, mitochondria also participate in key signaling events regulating cell proliferation and death [27]. PKB has a critical role in regulating apoptosis [28-30] and directly phosphorylates and interacts with key factors involved apoptosis signaling [30,31]. PKB also indirectly regulates apoptotic transcription factors such as FOXO3a and NFκB [30]. Additionally, activated PKB is localized to various subcellular compartments including the mitochondria and nucleus [32]. Therefore, there is a complex interplay between PKB signaling and mitochondria-mediated apoptosis.

We provide the first evidence that CTMP, a negative regulator of PKB, localizes to the mitochondria in a MTS-dependent manner (Figure 3A and 3B). We found CTMP is located at the mitochondrial intermembrane space and/or matrix (Figure 2D–2F). In addition, we discovered CTMP is phosphorylated on Ser37/Ser38 (Figure 1) and phosphorylation on these residues inhibited CMTP mitochondrial localization (Figure 3C). Furthermore, adenovirus-mediated CTMP overexpression sensitized cells to staurosporine-stimulated apoptosis (Figure 3D–3F). Interestingly CTMP interacts with Hsp70, resulting the sequestration of Hsp70 from Apaf-1 (Figure 4). Taken together, these data suggest CTMP is a novel mitochondrial protein and is involved in apoptosis (Figure 4D).

Evidence suggest that CTMP negatively regulates PKB activity in v-*Akt *transformed cells [10], ciliary ganglion neurons [11], and *K*-ras-induced lung cancer model [12]. This observation is further supported by a recent report showing epigenetic down-regulation of CTMP transcription in malignant glioblastomas [13]. As previously suggested [10], CTMP is phosphorylated *in vivo *in response to pervanadate stimulation (Figure 1). In the current study, two serine residues on CTMP that are phosphorylated in response to pervanadate *in vivo *were identified (Figure 1). Ser37/Ser38 is located at the amino-terminus of CTMP, close to the putative MTS, as predicted by MitoProt II 1.0a4 [22]. These two seine residues are conserved in CTMP homologues from mouse (NP_03707.1), rat (NP_001020188.1) and dog (NP_001074103.1) but lower organisms such as *D. melanogaster*, *S. cerevisiae*, *S. pombe *or *C. elegans *do not appear to contain a gene for CTMP, suggesting CTMP is a relatively recent protein during evolution and the two identified serine residues might be important for its function. In addition, Ser37 was predicted by the motif prediction program (Prosite) to be phosphorylated by Casein kinase II. However, this possibility needs to be experimentally evaluated in the context of apoptosis and will provide detailed insight into CTMP regulation.

CTMP was detected in the membrane and cytosol fraction of LN229 cells [10]. Furthermore, two molecular species corresponding to CTMP were observed in different cell lines, suggesting post-translational modifications. In addition, CTMP associated with intracellular structures similar to membrane ruffles [10]. Recently, a nuclear pool of CTMP was detected in human pancreatic duct epithelial cells [33]. Consistent with recent reports [17], a mitochondrial pool of CTMP from HEK 293 cells dependent on its MTS was detected in our experiments (Figure 2 and 3). Furthermore, this mitochondrial CTMP localization was inhibited by phosphorylation (Figure 3C). Therefore, CTMP may have distinct roles in various subcellular compartments.

Mitochondrial localization is mediated by the MTS. In most cases, the MTS is present as a cleavable sequence at the N terminus, also called a pre-sequence [23]. Bioinformatic analysis of the CTMP sequence indicated that a potential MTS exists in CTMP at the N-terminus of protein. This predicted N-terminal MTS appears to be functional since an N-terminal 31-amino acid deletion-mutant of CTMP did not localize to the mitochondria (Figure 3A and 3B). N-terminal MTS of mitochondrial precursors are in most cases cleaved by the mitochondrial-processing peptidase (MPP) as soon as the cleavage sites reach the mitochondrial matrix [34,35]. Mutational studies show the R-2 or R-3 motif are important but not sufficient to direct cleavage by MPP [35]. Site-directed mutagenesis of the -2 or -3 arginine in different precursor molecules completely or partially inhibits processing [36,37]. Further studies are needed to interrogate if the R-2 motif of CTMP is important in MTS-mediated mitochondria localization.

The molecular pathways that mediate apoptosis are tightly regulated by a series of positive and negative signals, the balance of which determines whether or not cells commit suicide [38]. Recent evidence indicates that the coordinated interaction between Hsps and the components of apoptosis machinery may determine cellular susceptibility to damaging stresses [24]. Indeed, CTMP associate specifically Hsp70, but not Hsp90, in HeLa cells (Figure 4A and 4B). Furthermore, complex formation of Hsp70 and Apaf-1 was decreased in CTMP-infected cell compared to controls (Figure 4C), indicating why CTMP overexpression sensitized the cell to apoptosis induced by staurosporine (Figure 4D). Indeed, Miyawaki and colleagues recently reported that upregulation of CTMP in hippocampal neurons is required for ischemia-induced neuronal death [39]. Based on previous studies ([10,12,40], CTMP phosphorylation event occurs at the plasma membrane in response to activation of growth factor signaling, leading to the release of PKB. In the case of apoptosis, phosphorylation of the mitochondria pool of CTMP by as-yet-unidentified kinase will promote the exclusive cytoplasmic localization of CTMP, resulting the sequestration of Hsp70 from Apaf-1. One can also proposed that 14-3-3 may bind the phosphorylated CTMP and maintain it's cytoplasmic localization. In this regard, it will be of course interesting to discover how phosphorylation affects the interaction between CTMP and Hsp70. Further studies are required to elucidate mechanisms of CTMP-dependent apoptosis under pathophysiological conditions.

## Conclusion

In summary, this work provides the first evidence for the involvement of phosphorylation in the MTS-mediated mitochondrial localization of CTMP. In addition to its role in PKB inhibition, CTMP has a key role in mitochondria-mediated apoptosis by localizing to mitochondria. Whether phosphorylation of these serine residues affects CTMP protein-protein interactions remains unknown. Further analysis is likely to elucidate additional CTMP regulatory mechanisms, which will provide a better understanding of its role in signaling and its potential in therapeutic applications.

## Methods

### Reagents

The anti-CTMP antibody used was described previously [10]. Antibodies were purchased from the following companies: anti-AIF and anti-COX IV (Cell Signaling), anti-GFP (Santa Cruz Biotech), anti-PARP (BD Biosciences), anti-Flag (Sigma), HRP-conjugated anti-mouse IgG (Calbiochem), HRP-conjugated anti-rabbit IgG (Calbiochem), and HRP-conjugated anti-sheep IgG antibody (Sigma). Staurosporine and sodium orthovanadate (Na_3_VO_4_) were purchased from Sigma.

### Construction of expression vectors

GFP-N-terminal-tagged CTMP (GFP-NT-CTMP) was previously reported [10]. GFP-C-terminal-tagged CTMP (GFP-CT-CTMP) was subcloned into pEGFP-N3 (Clonetech). CTMP deletion mutants were constructed in pEGFP-N1 vector (GFP-CT-CTMP-ΔN31). Ser37/Ser38 negatively charged side group-mimic mutants (S37D/S38D) were created by using the QuikChange Site-Directed Mutagenesis Kit (Stratagene), using pEGFP-N3-CTMP as a template. An adenoviral CTMP vector in pENTR-3C was prepared using the Adenoviral Expression Kit (Invitrogen). Adenoviruses were purified as described previously [41]. Constructs were confirmed by DNA sequencing. Mutagenic and cloning oligonucleotide sequences are available upon request.

### Cell culture and stimulation

U2OS, CCL64, HeLa, and HEK 293 cells were maintained in Dulbecco's modified Eagle's medium (DMEM) supplemented with 10% FBS, 2 mM glutamine, 100 unit/ml penicillin, and 100 μg/ml streptomycin (Life Technologies) and were transfected using FuGene 6 (Roche), jetPEI (Q-biogene), or Lipofectamine (Invitrogen) reagent. The transfection mixture was removed after 24 h and cells were serum-starved for 16 h before stimulation for 15 min with 100 μM pervanadate, prepared in 0.2 mM H_2_O_2 _[42]. To induce apoptosis, cells were treated with 1 mM staurosporine for the indicated time.

### *In vivo *labeling of stable CCL64 cells expressing wild type Flag-CTMP and phosphoamino acid analysis

Metabolic labeling of CCL64 cells was performed as described previously, with minor modifications [43]. Briefly, cells were stimulated with buffer or 100 μM pervanadate for 15 min. The ^32^P-labeled band corresponding to Flag-CTMP was excised, reduced, alkylated, and cleaved with 1 μg of trypsin (Promega, sequencing grade). Phosphoamino acids were identified following hydrolysis in 6 M HCl containing 0.1 mg/ml bovine serum albumin at 110°C for 60 min. Hydrolysates were separated by thin layer electrophoresis at pH 3.5 to resolve phosphoserine (pS), phosphothreonine (pT), and phosphotyrosine (pY) [44]. Radioactivity was detected using a PhosphoImager (Molecular Dynamics).

### Phosphorylation site mapping by mass spectrometry

Extracted peptides were analyzed by high-performance liquid chromatography (HPLC) interfaced with electrospray ionization mass spectrometry (ESI-MS), using a Rheos 4000 chromatograph equipped with a 1 × 250 mm Vydac (Hesperia) C18 column and interfaced with a Sciex API 300 mass spectrometer (PE Sciex), operated in the single quadrupole mode on Q1. Analysis of mass spectra of phosphopeptides was performed as described previously [43]. Mass spectra were acquired on a Sciex API 300 triple quadrupole mass spectrometer equipped with a NanoESI source (Protana).

### Confocal imaging analysis of CTMP localization

U2OS cells were grown on glass coverslips and transfected with GFP-CTMP or pDsRed-Mito, a mitochondrial marker. After 24 h, cells were fixed in 4% paraformaldehyde at room temperature for 10 min, mounted with Vectashield (Vector Laboratories) and visualized using a OLYMPUS 510 confocal microscope. Differential localization of CTMP (Cyt: cytoplasm, PM: plasma membrane, Mit: mitochondria, Nuc: Nucleus) was examined using confocal microscopy. At least 200 cells were counted from three distinct fields for each transfected group.

### Preparation of mitochondria and plasma membranes

Cells were washed with PBS and resuspended in mitochondrial fraction buffer (20 mM HEPES, pH 8.0, 10 mM KCl, 1.5 mM MgCl_2_, 1 mM EDTA, 250 mM sucrose, 1 mM PMSF, 10 g/ml leupeptin, 10 g/ml aprotinin, and 0.2 mM sodium orthovanadate) for 30 min on ice and then homogenized. Unbroken cells and nuclei were removed. The supernatant was centrifuged at 10,000 × g for 30 min at 4°C. The resultant supernatant, representing the cytosolic fraction, was centrifuged at 100,000 × g for 1 h at 4°C. The resultant pellet was washed with 500 μl of mitochondrial fraction buffer and solubilized in lysis buffer to generate the mitochondrial fraction. Isolated mitochondrial fractions were further treated with 2 M sodium chloride, 100 mM sodium carbonate (pH 11.2), or 1% Triton X-100 for 30 min. Samples were ultracentrifuged at 100,000 × g for 1 h to separate the supernatant (S) and precipitate (P) fractions.

## Competing interests

The authors declare that they have no competing interests.

## Authors' contributions

LP and YL participated in the design of the study, carried out bench experiments and drafted the manuscript. SMM provided the initial information for the identification of phosphorylation sites of CTMP. KJY, KAP, HSB and MW helped carrying out bench experiments related to this study. JP, RS and DH carried out mass spectrometric analysis for the identification of phosphorylation site. JH, JLK, GRK, GMH and JHS provided intellectual input in drafting of the manuscript. JYC and TC provided material for this study and helped drafting the manuscript by providing critical intellectual input. DPB and BAH participated in the design of the study, provided material for this study and helped drafting the manuscript. JP designed this study, interpreted the results, helped drafting the manuscript, and finalized the manuscript after input from other authors. All authors read and approved the final manuscript.
